# DNA methylation modulates epigenetic regulation in colorectal cancer diagnosis, prognosis and precision medicine

**DOI:** 10.37349/etat.2024.00203

**Published:** 2024-01-28

**Authors:** Jingxin Ye, Jianfeng Zhang, Weifeng Ding

**Affiliations:** Chinese Peoples Liberat Army China PLA General Hospital, China; ^1^Department of Laboratory Medicine, Affiliated Hospital of Nantong University, Nantong 226001, Jiangsu Province, China; ^2^Department of Gastroenterology, Affiliated Hospital of Nantong University, Nantong 226001, Jiangsu Province, China; ^3^Department of Gastroenterology, The Affiliated Suqian Hospital of Xuzhou Medical University, Suqian 223800, Jiangsu Province, China

**Keywords:** Epigenetic modification, DNA methylation, colorectal cancer, diagnosis, metabolism reprogramming

## Abstract

Colorectal cancer (CRC) is a multifaceted disease influenced by the interplay of genetic and environmental factors. The clinical heterogeneity of CRC cannot be attributed exclusively to genetic diversity and environmental exposures, and epigenetic markers, especially DNA methylation, play a critical role as key molecular markers of cancer. This review compiles a comprehensive body of evidence underscoring the significant involvement of DNA methylation modifications in the pathogenesis of CRC. Moreover, this review explores the potential utility of DNA methylation in cancer diagnosis, prognostics, assessment of disease activity, and prediction of drug responses. Recognizing the impact of DNA methylation will enhance the ability to identify distinct CRC subtypes, paving the way for personalized treatment strategies and advancing precision medicine in the management of CRC.

## Introduction

As a malignant tumor affecting the digestive system, colorectal cancer (CRC) remains one of the leading causes of cancer-related mortality worldwide. According to projections by the American Cancer Society, there were approximately 151,030 new CRC cases (8.0% of all cancers) and 52,580 CRC-related deaths (8.5% of all cancer-related deaths) in the USA in 2022 [1]. According to the latest release of national cancer statistics by the China Cancer Center in February 2022, the estimated number of new cases of malignant tumors in China in 2016 was 4,064,000, with 408,000 cases of CRC, making it the second most common type after lung cancer. The estimated number of deaths from malignant tumors nationwide is 2,413,500, with CRC causing 195,600 deaths and ranking fifth in terms of mortality rate.

The pathogenesis of CRC is primarily determined by epigenetics, defined as hereditary changes in gene expression without permanent alterations in the DNA sequence [2]. Among various epigenetic modifications, DNA methylation stands out as the most significant mode of variation, with CRC epigenetic instability primarily manifested through abnormal DNA methylation and genome-wide DNA demethylation in the promoter region and the 5-terminal regulatory region. This ubiquitous epigenetic modification is closely associated with gene expression, with 5-methylcytosine (5mC) and 5-hydroxymethylcytosine (5hmC) serving as key epigenetic hallmarks [3–5]. It is well-established that DNA methyltransferases (DNMTs) catalyze the addition of a methyl group (CH_3_) at the C5 position of the cytosine ring, representing the most well-understood DNA methylation process. Imbalances in genomic methylation significantly contribute to carcinogenesis [6].

The conventional progression pattern of CRC involves the transition from normal intestinal mucosa to early adenomatous polyps, advanced adenomatous polyps, and ultimately to CRC [3–5]. To capture this progression, we collected biomarkers spanning from normal mucous membranes to proliferative polyps, adenomas, and carcinomas. Additionally, non-invasive methylation biomarkers for monitoring CRC patients have been summarized. Examples include markers associated with blood or feces closely linked to DNA methylation, serving as potential biomarkers for diagnosing or prognosis prediction of CRC. Moreover, the metabolic reprogramming of cancer cells during malignant transformation has become a research hotspot, offering new therapeutic targets for CRC treatment. In summary, epigenetic modules provide a potential interplay through which genetic and environmental risk factors intersect, contributing to the susceptibility and etiopathogenesis of CRC.

## Candidate diagnostic DNA methylation biomarkers in CRC

Despite previous studies on epigenetic biomarkers in CRC, over 100 new studies have emerged since the most recent comprehensive review. Herein, we focused on molecular markers associated with DNA methylation, especially studies involving non-invasive analysis.

### Biomarkers associated with normal surrounding mucosa, polyp, and CRC tissue

It is now understood that early diagnosis and risk assessment of CRC can significantly reduce mortality. As our understanding of CRC development advances, more studies have focused on polyps and normal surrounding mucosa for early diagnosis. These have potential use in pathology sections combined with methylation testing for patients undergoing biopsy during colonoscopy, further assessing the risk of carcinogenesis.

For example, secreted frizzled related protein-1 (*SFRP1*) and *SFRP2* have been extensively researched for their roles in CRC. The methylation levels of *SFRP1*, *SFRP2*, and Wnt inhibitory factor-1 (*WIF1*) in tumor tissues were found to be significantly upregulated compared to adjacent non-neoplastic tissues. It is widely believed that the hypermethylation of the *SFRP2* promoter and co-hypermethylation of *SFRP1* and *SFRP2* may serve as independent prognostic predictors of survival advantage in CRC patients post-surgery. Additionally, methylated *SFRP2* has been identified as a non-invasive biomarker for CRC detection, providing diagnostic value and the potential to predict tumor risk in surrounding normal mucosa [7–9].

In a study using pyrosequencing, CRC and adenomatous polyp samples from cancer patients were compared to normal tissues from healthy donors. The peritumoral benign mucosa of cancer patients showed relatively high frequencies and levels of death-associated protein kinase (*DAPK*), O^6^-methylguanine DNMT (*MGMT*), and tissue factor pathway inhibitor-2 (*TFPI2*) methylation compared to normal mucosa from healthy volunteers. Further investigations are needed to determine the clinical value of these genes, particularly *MGMT*, whose promoter methylation is present at the early stages of tumorigenesis and can also be measured in normal mucosa. Notably, *MGMT* methylation has been associated with a good prognosis and could predict the response to dacarbazine in a phase II clinical study for metastatic CRC (mCRC) [10, 11].

Moreover, the expression levels of C-X-C motif chemokine receptor 4 (*CXCR4*) were significantly elevated in tumor stromal cells and tumor colonocytes compared to paired adjacent normal mucosa. Additionally, there were notable differences in CXCR4 protein expression between microsatellite instable (MSI) and microsatellite stable (MSS) tumor cell lines. Although no differential methylation was detected in *CXCR4* compared to adjacent mucosa, the accumulation of 5hmC was observed in the *CXCR4* gene bodies in CRC [12, 13]. Further studies are warranted to understand the mechanism of *CXCR4* promoter hypermethylation and its role in CRC. The above research findings demonstrate that DNA methylation may serve as a predictive tool for assessing the risk of cancer in normal surrounding mucosa.

When considering polyps, it is imperative to address serrated polyps, constituting approximately 15% to 30% of all CRC cases [14]. Serrated polyps are primarily distinguished by *BRAF* and/or *KRAS* genetic mutations, coupled with epigenetic alterations in the CpG island methylator phenotype (CIMP), collaborating to initiate and propel malignant transformation from normal colon mucosa to polyps and ultimately to CRC [15]. The methylation patterns of numerous genes exhibit variation from normal mucous membranes to proliferative polyps, adenomas, and carcinomas. For instance, caudal type homeobox 2 (*CDX2*) has been firmly established as a diagnostic biomarker for CRC, and the loss of *CDX2* has emerged as an independent adverse prognostic factor associated with molecular features of the serrated pathway involving promoter methylation and histone deacetylation [16]. Notably, sessile serrated lesions have been identified as pivotal precursor lesions for the CpG island-methylated pathway leading to CRC. Genome-wide methylation profiling has revealed a novel differentially methylated biomarker, metallophosphoesterase domain containing 2 (*MPPED2*), demonstrating methylation alterations across the progression from normal mucosa to hyperplastic polyps, adenomas, and carcinomas. This finding suggests sequential epigenetic variations in the *MPPED2* promoter region during colorectal tumorigenesis, holding promise as a biomarker for early diagnosis and stage surveillance of colorectal neoplastic progression [17]. Recent research has also revealed the high accuracy of secretin receptor (*SCTR*) methylation in detecting both CRC and adenomas [18]. Moreover, investigations into the cellular origin, molecular heterogeneity, and immunogenicity of polyp precursors may yield insights into the diagnosis and therapy of CRC. Single-cell transcriptome and imaging maps of human colorectal polyps provide a framework for precise monitoring and prevention of CRC [19]. In a recent study, cells were collected from 48 polyps, 27 normal tissues, and 6 CRC samples, generating a single-cell chromatin accessibility profile and a single-cell transcriptome [20]. Adenomatous polyps exhibited a remarkably consistent epigenetic and transcriptional trajectory during progression to CRC. DNA methylation changes in sporadic CRC demonstrated a strong negative correlation with accessibility alterations along this continuum, further identifying regulatory biomarkers for polyp molecular staging [19].

Several multigene panels have undergone clinical validation, as exemplified by a study involving 523 tissue samples encompassing CRC, adenoma, and normal colonic mucosa. This investigation comprehensively analyzed six biomarkers through quantitative methylation, revealing a novel epigenetic biomarker panel with exceptional sensitivity and specificity for both CRC and adenoma. Notably, hypermethylation of the promoters of genes cannabinoid receptor interacting protein 1 (*CNRIP1*), fibrillin 1 (*FBN1*), internexin neuronal intermediate filament protein, alpha (*INA*), myelin and lymphocyte (*MAL*), synuclein, alpha (*SNCA*), and spastic paraplegia 20 (*SPG20*) was prevalent in CRC (65% to 94%) and adenoma (35% to 91%), whereas normal mucosal samples exhibited minimal methylation (0% to 5%). The combined sensitivity of testing positive for at least two of the six biomarkers was notably high, reaching 94% and 93% in CRC and adenoma samples, respectively, with an impressive specificity of 98% [21]. In another study, a different biomarker panel analyzed methylation in corresponding fresh-frozen tissue samples, indicating rates of 27% for adenomatosis polyposis coli (*APC*; specificity = 97%), 39% for *MGMT* (specificity = 96%), 58% for ras association domain family member 2a (*RASSF2A*; specificity = 100%), and 74% for *WIF1* (specificity = 98%) [22]. These biomarker panels exhibit suitability for early tumor detection. A comprehensive summary of tissue-based diagnostic marker candidates for CRC is provided in Table 1.

**Table 1:** Summary of evidence for most promising methylation biomarkers for diagnosis of CRC in tissue (sensitivity > 75%)

**Biomarker gene(s)**	**Sensitivity (%)**	**Specificity (%)**	**Reference**
*AKR1B1*, *SEPT9*	98	99	[23]
*APC*, *MGMT*, *RASSF2A*, *WIF1*	87	92	[22]
*APC*, *p16^INK4a^*, *MGMT*, *RARB2*	77	100	[24]
*BMP3*	84	95	[25]
*CDH1*	87	74	[26]
*CMTM3*, *MDFI*, *SSTR2*	81	91	[27]
*CNRIP1*, *FBN1*, *INA*, *MAL*, *SNCA*, *SPG20*	94	98	[21]
*GDNF*, *SNAP91*, *NDRG4*	86	96	[28]
*EFHD1*	79	78	[29]
*ITGA4*	89	88	[30]
*NDRG4*	81–92	92–95	[25, 31]
*MGMT*, *RASSF1A*, *SEPT9*	97	74	[11]
*SEPT9*	78–97	96–97	[32, 33]
*SFRP1*	93	100	[34]
*SFRP2*	83–91	100	[35, 36]
*SPG20*	94	99	[21]
*TFPI2*	99	94	[10]
*VIM*	86	95	[37]

*AKR1B1*: aldo-keto reductase family 1 member B; *SEPT9*: septin 9; *RARB2*: retinoic acid receptor beta 2; *BMP3*: bone morphogenetic protein 3; *CDH1*: cadherin 1; *CMTM3*: CKLF like MARVEL transmembrane domain containing 3; *MDFI*: myoD family inhibitor; *SSTR2*: somatostatin receptor 2; *GDNF*: glial cell derived neurotrophic factor; *SNAP91*: synaptosome associated protein 91; *NDRG4*: NDRG family member 4; *EFHD1*: EF-hand domain family member D1; *ITGA4*: integrin subunit alpha 4; *VIM*: vimentin

### Biomarkers in plasma

In recent years, extensive investigations have sought to ascertain the efficacy of biomarkers in early CRC diagnosis using blood samples. Specifically, ten hypermethylated CpG sites within three genes, namely chromosome 20 open reading frame 194 (C20orf194), LIF receptor subunit alpha (*LIFR*), and zinc finger protein 304 (*ZNF304*), were identified as CRC-specific markers. A random forest model assessing the accuracy of these ten markers in predicting CRC across three independent datasets yielded a range of 85.7% to 94.3%. The hypermethylation of these markers was discerned in cell-free DNA (cfDNA) samples from CRC patients. In the cfDNA validation cohort (*N* = 155), the biomarker panel demonstrated a sensitivity of 69.5% and specificity of 91.7%. The hypermethylation of these ten CpG sites exhibited specificity to CRC tissues and holds promise as non-invasive cfDNA markers for CRC diagnosis [38].

A decade ago, long interspersed element-1 (*LINE-1*) was identified as a prognostic biomarker, with plasma hypomethylation associated with increased tumor size, more frequent distant metastases, and elevated cancer-specific mortality [39, 40]. The tumor suppressor gene (*TSG*), IKAROS family zinc finger 1 (*IKZF1*), has demonstrated differential methylation in CRC through multivariate bisulfite sequencing. Notably, it exhibited minimal methylation levels in peripheral blood DNA, suggesting its potential as a blood-based diagnostic marker [41, 42]. However, current diagnostic techniques exhibit limited sensitivity for early CRC detection.

One promising biomarker, *SEPT9* methylation, has exhibited robust diagnostic performance with a high cancer detection rate (57–64%) in CRC stage 0 and stage I patients [43, 44]. Methylation of branched chain amino acid transaminase 1 (*BCAT1*) and *IKZF1* has been recurrently observed in CRC, with nearly all cancer tissues displaying significant methylation levels of these two genes. In a case-control study involving 218 individuals, the combined analysis of *IKZF1* and *BCAT1* demonstrated a diagnostic sensitivity of 77% for CRC detection, with detection rates of 50% and 68% for stages I and II, respectively.

While plasma-based *SEPT9* methylation has been commercialized for clinical use [45, 46]. the need persists for methylation-based biomarkers capable of simultaneously diagnosing CRC and its precursor lesions. Despite the high specificity demonstrated by plasma SEPT9 methylation markers in large-scale studies, their sensitivity for detecting precursor lesions remains limited [43, 44]. Recent research has identified the methylation profiles of candidate zinc finger genes (ZFGs) as potential biomarkers for early CRC diagnosis, particularly in KRAS-mutated patients. Two of these *ZFGs*, estrogen receptor 1 [*ESR1*; sensitivity 78%, specificity 97%, area under the curve (AUC) = 0.91] and *ZNF132* (sensitivity 83%, specificity 97%, AUC = 0.93), displayed significantly higher diagnostic capabilities than *SEPT9* (sensitivity 83%, specificity 87%, AUC = 0.91) [47].

Additionally, carcinoembryonic antigen (*CEA*) is the sole blood test recommended by guidelines for monitoring CRC recurrence, but its sensitivity and specificity are suboptimal. Comparative studies between methylated *BCAT1* and *IKZF1* DNA blood tests and *CEA* for recurrent CRC have shown that *BCAT1*/*IKZF1* exhibits higher sensitivity for recurrence detection, with a probability of positive recurrence twice that of *CEA* [48–50].

Furthermore, comprehensive methylation analysis has unveiled aberrant methylation of CpG sites in syndecan 2 (*SDC2*) in tumor tissues of most CRC patients, demonstrating a huge potential for quantifying blood *SDC2* methylation for early CRC detection [35]. Moreover, a stool DNA-based *SDC2* methylation test using linear target enrichment quantitative methylation-specific real-time polymerase chain reaction (PCR) has shown promise for early CRC detection with high specificity [51]. Methylation of promoter sequences in *SDC2*, *SFRP1*, *SFRP2*, and proline rich membrane anchor 1 (*PRIMA1*) was observed in 85.1%, 72.3%, 89.4%, and 80.9% of plasma samples from CRC patients, respectively. When applied as a panel, these methylation markers distinguished CRC patients from controls with 91.5% sensitivity and 97.3% specificity [52].

Another frequently cited non-invasive methylation marker for CRC diagnosis is the methylation of the *VIM* gene. In plasma samples, *VIM* methylation has demonstrated a sensitivity of up to 59% and a specificity of 93%, exhibiting notably heightened sensitivity at advanced disease stages. Furthermore, the combination of *SFRP2* and *VIM* methylation has yielded increased sensitivity and specificity in the detection of CRC. However, *VIM* methylation has been found to be even more sensitive and specific in feces and urine samples [53, 54]. The summarized information is presented in Table 2.

**Table 2:** Summary of evidence for most promising methylation biomarkers for diagnosis of CRC in blood (sensitivity > 75%)

**Biomarker gene(s)**	**Sensitivity (%)**	**Specificity (%)**	**Reference**
*ALX4*	80–83	41–70	[55, 56]
*ALX4*, *BMP3*, *NPTX2*, *RARB*, *SDC2*, *SEPT9*, *VIM*	91	73	[57]
*ALX4*, *SEPT9*, *TMEFF2*	81	90	[32, 55]
*APC*, *MGMT*, *RASSF2A*, *WIF1*	87	92	[22]
*BCAT1*, *IKZF1*	66–77	92–94	[58]
*C9orf50*, *CLIP4*, *KCNQ5*	85	99	[59]
*C9orf50*, *KCNJ12*, *ZNF132*, *TWIST1*	80	97	[60]
*CYCD2*, *HIC1*, *PAX5*, *RASSF1A*, *RB1*, *SRBC*	84	68	[61]
*EFHD1*	79	78	[29]
*EFDH1*, *PPP1R3C*	90	64	[29]
*NPY*	87	80	[62]
*PPP1R3C*	79–81	81	[29]
*PRIMA1*	81	73	[49]
*PRIMA1*, *SDC2*, *SFRP1*, *SFRP2*	92	97	[49]
*RASSF2*	93	53	[61]
*SDC2*	87–89	95–97	[49, 50]
*SDC2*, *SEPT9*	86–89	87–93	[63–66]
*SDC2*, *SEPT9*, *SFRP2*	94	89	[67]
*SEPT9*	75–90	92–97	[32, 33, 68–72]
*SEPT9*, *CEA*	86	96	[73]
*SFRP1*	80	92	[74]
*SPG20*	81	97	[75]

*ALX4*: ALX homeobox 4; *NPTX2*: neuronal pentraxin 2; *TMEFF2*: transmembrane protein with EGF like and two follistatin like domains 2; *KCNJ12*: potassium inwardly rectifying channel subfamily J member 12; *TWIST1*: twist family bHLH transcription factor 1; *CYCD2*: predicted protein; *HIC1*: HIC ZBTB transcriptional repressor 1; *PAX5*: paired box 5; *RB1*: RB transcriptional corepressor 1; *SRBC*: CD2 molecule; *PPP1R3C*: protein phosphatase 1 regulatory subunit 3C; *NPY*: neuropeptide Y

### Biomarkers in stool and urine

At present, three methylation biomarkers—*NDRG4*, *BMP3*, and *SEPT9*-have received Food and Drug Administration (FDA) approval for CRC screening, representing the inaugural endorsement of a fecal-based multitarget group for such screening [76]. Another testing marker, *VIM* methylation, is commercially available but awaits FDA approval [77]. In stool samples, methylation of the *VIM* gene has exhibited a sensitivity of 81% and a specificity of 95% [54]. Additionally, a study noted the presence of *VIM* methylation in urine samples in 75% of CRC cases [53].

It has been reported that the methylation levels of the *TFPI2* promoter and most CpG sites in CRC exceeded those in normal tissues [78]. In stool samples, the positive detection rate was notably higher, reaching 93.4% for CRC and 81.3% for adenoma, compared to normal samples, with a specificity of 94.3%. In another investigation, *TFPI2* methylation in the feces of stage I to III CRC patients emerged as a potential biomarker for early CRC detection, demonstrating a sensitivity range of 76% to 89% and a specificity range of 79% to 93% [10, 79]. The incorporation of *TFPI2* methylation in fecal DNA may refine noninvasive CRC screening strategies.

A study validated the methylated panel comprising *COL4A1*, *COL4A2*, *TLX2,* and *ITGA4* using 240 CRC stool samples. The detection accuracy for CRC ranged from 82.5% to 92.5%, while for adenomas ≥ 1 cm, it varied from 41.6% to 58.4%, with specificities ranging between 88.0% and 96.4%. The combination of *COL4A2* and *TLX2* in feces exhibited the best performance, detecting 91.3% of CRC cases and 51.9% of advanced adenomas, with a specificity of 97.6% [80].

The TSG, *NDRG4*, is downregulated in CRC [81]. An assessment of methylated *NDRG4* in urine samples revealed a sensitivity of 73% for CRC, although this did not surpass the 76% sensitivity achieved by stool-based assays. Moreover, *NDRG4* methylation assays have been explored in tissue and plasma [82, 83]. These details are summarized in Table 3.

**Table 3:** Summary of evidence for most promising methylation biomarkers for diagnosis of CRC in stool (sensitivity > 75%)

**Biomarker gene(s)**	**Test**	**Sensitivity (%)**	**Specificity (%)**	**Reference**
*APC*, *ATM*, *hMLH1*, *HLTF*, *MGMT*, *SFRP2*	CRC	75	90	[84]
*COL4A2*, *TLX2*	CRC	91	98	[80]
*FBN1*, *SNCA*	CRC	84	93	[85]
*GATA5*	CRC	84	83	[86]
*GDNF*, *SNAP91*, *NDRG4*	CRC	86	96	[28]
*HPP1*	CRC	100	71	[87]
*HPP1*, *MGMT*, *SFRP2*	Adenoma	94	77	[87]
*ITGA4*, *SFMBT2*, *THBD*, *ZNF304*	CRC	96	87	[88]
*KCNJ12*, *VAV3-AS1*, *EVC*	CRC	83	71	[88]
*NDRG4*	CRC	76–98	89	[82, 83, 86]
*NDRG4*, *BMP3*	CRC	98	90	[89]
*NDRG4*, *BMP3*, mutation *KRAS*, hemoglobin	CRC	92–98	87–90	[89, 90]
	Adenoma	82	93	[54]
*NEUROD1*, *FAM72C*	CRC	83	77	[88]
*MLH1*, *VIM*, *MGMT*	CRC	75	86.5	[91]
*RARB2*, *p16^INK4a^*, *MGMT*, *APC*	CRC	75	/	[24]
*SDC2*	CRC	90	89	[51]
*SFRP2*	Adenoma	76–87	55–100	[9, 52]
*SFRP2*, *TFPI2*, *NDRG4*, *BMP3*	CRC	94	55	[52]
*SEPT9*	Adenoma	83	92	[92]
*VIM*, *NDRG4*, *BMP3*, *TFPI2*	CRC	89	90	[76]
*SFRP1*	CRC	89	86	[93]
*VIM*	CRC	38–81	82–95	[94]
	Adenoma	33–83	93–100	[95]
*SFRP2*, *GATA4/5*, *NDRG4*, *VIM*	CRC	96	65	[96]
*TFPI2*	Adenoma	81	94	[97]
	CRC	76–93	89–100	[97, 98]

*ATM*: ATM serine/threonine kinase; *hMLH1*: homo sapiens DNA mismatch repair; *HLTF*: helicase like transcription factor; *GATA5*: GATA binding protein 5; *HPP1*: hyperpigmentation progressive 1; *SFMBT2*: Scm like with four mbt domains 2; *THBD*: thrombomodulin; *VAV3-AS1*: VAV3 antisense RNA 1; *EVC*: EvC ciliary complex subunit 1; *NEUROD1*: neuronal differentiation 1; *FAM72C*: family with sequence similarity 72 member C; *MLH1*: MutL homolog 1

## Candidate DNA methylation biomarkers for therapy and therapy-response

In the realm of CRC treatment, significant emphasis is placed on identifying biomarkers for effective tumor detection and understanding tumor suppressor factors that can mitigate the proliferation, migration, and invasion capabilities of CRC cells. The appeal of epigenetic alterations as therapeutic targets stems from their potential reversibility. However, challenges arise in clinical scenarios where certain patients fail to respond to chemotherapy, necessitating the identification of biomarkers to discern non-responders and mitigate adverse effects associated with chemotherapy. Insights from the literature have culminated in the identification of potential molecular markers for CRC treatment. For instance, in KRAS mutant CRC cells, solute carrier family 25 member 22 (*SLC25A22*) has been identified as a promoter of DNA methylation expression. Disrupting this pathway by knocking out SLC25A22-induced DNA demethylation, re-activating protocadherins. This, in turn, suppressed WNT/β-catenin signaling, stem cell characteristics, and resistance to 5-fluorouracil [99]. Although confined to cell lines, human tissue samples, and animal models, this pathway holds significant potential for CRC treatment and potentially extends to other cancers.

Several predictive DNA methylation biomarkers, though not clinically approved, warrant further evaluation in clinical trials [100]. In this respect, *LINE-1* elements, constituting 17% of the human genome, undergo hypomethylation associated with genome-wide hypomethylation, correlating with early-onset CRC and poor prognosis. *LINE-1* methylation has emerged as a therapeutic marker, correlating with the prognosis of patients with stage II or III CRC undergoing oral fluoropyrimidines therapy [101].

Additional research underscores the involvement of DNA methylation in the formation of tumor-reinvocative bystander CD8^+^ tumor-infiltrating lymphocytes (TILs). Zou et al. [102] developed a quantitative DNA methylation-based signature to evaluate CD8^+^ TILs, offering a valuable resource for developing novel methylation biomarkers and identifying potential druggable targets.

Previous investigations have established the downregulation of *DMTN* expression in CRC tissues. Overexpression of *DMTN* has shown efficacy in inhibiting the invasion and metastasis of CRC cells, indicating its potential as a therapeutic target for precision medicine in CRC patients [103]. The heat shock protein 90 (*HSP90*) inhibitor, ganetespib, has demonstrated effectiveness in modulating DNA methylation by down-regulating the expression of DNMTs, which are positively correlated with global DNA methylation levels in CRC cell lines. Ganetespib presents a promising approach to modulating DNA methylation and promoting the expression of silenced genes in CRC [104]. Ubiquitin like with PHD and ring finger domains 1 (*UHRF1*) depletion, coupled with histone deacetylase domain protein (*HDAC*) inhibition, has been shown to induce rapid DNA demethylation, reviving silenced genes and significantly suppressing CRC cell proliferation. This dual targeting of *UHRF1* and *HDAC* has emerged as a potential and effective therapeutic strategy for CRC [105]. High *UHRF1* levels, coupled with low TSG expression, are negatively associated with CRC progression and reduced patient survival, suggesting the need to explore critical *UHRF1* domains and their relevance to CRC prognosis, pointing toward novel therapeutic avenues [106].

The deficient mismatch repair (*dMMR*) of colorectal tumors is significantly correlated with CIMP status and dMMR is a predictive marker for the lack of efficacy of 5-fluorouracil-based adjuvant chemotherapy [57, 107, 108]. A study evaluates the role of 5-azacytidine in increasing sensitivity in refractory CIMP-high patients receiving capecitabine and oxaliplatin chemotherapy [109]. The preclinical results of this study are encouraging, but there is still a lack of sufficient clinical evidence that epigenetic therapies resensitize chemotherapy [110]. Research on epigenetic therapies to reprogram tumor cells to re-sensitize them to radiation and cytotoxic therapies is very promising. DNMT and HDAC inhibitors can re-express TSGs such as *p16*, *RASSF1A*, *DAPK*, and methylated genes involved in specific chemotherapeutic pathways [111–114]. Thus, “reprogramming” tumor cells can sensitize them to cytotoxic agents. One study showed that MSS-cell lines were more likely to show chemosensitization to irinotecan after pretreatment with 5-azacytidine [115].

Identification of methylation markers in CRC has proven valuable in monitoring treatment response and guiding adjustments to current treatment regimens based on the patient’s methylation phenotype. For instance, *MGMT* hypermethylation in advanced rectal cancer patients treated with 5-fluorouracil and dacarbazine was associated with a favorable prognosis and improved response to neoadjuvant radiotherapy [116]. Another study linked HPP1 methylation in mCRC to the efficacy of treatment with bevacizumab, fluoropyrimidine, and oxaliplatin, revealing that patients with undetectable methylated *HPP1* levels responded better to treatment [117]. Recent research confirmed the utility of methylation markers, including boule homolog (*BOLL*), DCC netrin 1 receptor (*DCC*), and *SFRP2*, for monitoring neoadjuvant chemotherapy response in CRC patients with liver metastases [118, 119].

Furthermore, combination of epigenetic therapy and immunotherapy for CRC is very promising. Candidate DNA methylation markers for prediction can reduce resistance to therapies and evaluate patient suitability for targeted therapy [120]. Epigenetic modifiers can alter the immunogenicity of the tumor microenvironment and enhance the effects of immunotherapy and immune checkpoint inhibitors [121]. The addition of entheogens or azacitidine to anti-cytotoxic T lymphocyte-associated protein 4 (CTLA-4) and anti-programmed cell death 1 (PD-1) inhibitors significantly inhibited the development of CRC, and the combination therapy was superior to either class of drugs alone [122]. In clinical trials, pembrolizumab has shown effective therapy responses in many patients with microsatellite stability MSI-H cancer. However, in the case of MSS CRC, immunotherapy has shown little to no effect [123, 124]. Therefore, distinguishing subgroups and overcoming the inefficacy of MSS CRC subgroups is clinically important.

## Candidate DNA methylation markers for prognosis prediction

Circulating tumor DNA (ctDNA) has emerged as a valuable diagnostic marker in various cancers. Epigenetic alterations are dysregulated in cancer and can be detected in liquid biopsies, such as effusion, urine, stool, and blood. Many of these epigenetic markers are reliable for CRC screening and serve as poor prognostic indicators. Epigenetic biomarkers offer the potential to monitor cancer progression, treatment response, and recurrence throughout the entire continuum of cancer care. However, prognostic DNA methylation biomarkers for clinical practice, particularly in patients requiring chemotherapy, remain scarce. Potential markers such as *ESR1*, *ZNF132*, and cytoplasmic polyadenylation element binding protein 1 (*CPEB1*) hold promise for serving as prognostic and predictive markers for CRC [47, 96].

In a prospective cohort study involving a high-risk population of 1,493 participants, researchers confirmed the efficacy of a single ctDNA methylation biomarker, cg10673833, in achieving high sensitivity (89.7%) and specificity (86.8%) for detecting CRC and precancerous lesions. This underscores the significance of ctDNA methylation biomarkers in CRC surveillance and prognosis [125]. Additionally, shore methylation of MLH1, irrespective of genotype, was found to be unrelated to promoter CpG island hypermethylation or MSI status [126]. However, the prognostic value of CIMP in CRC patients remains inconclusive in current literature, with CIMP representing a distinct pathway in CRC development associated with chromosomal stability (MSS) and a low mutant rate of adenomatous polyposis coli, both linked to taxane resistance [97].

Significant involvement of CpG island hypermethylation in the widespread reduction of protocadherin beta 3 (*PCDHB3*) levels was observed. Previous studies have identified *PCDHB3* as a novel TSG in CRC, inhibiting the nuclear factor kappa-B (NF-κB) pathway. Consequently, the expression and localization of PCDHB3 are considered prognostic biomarkers for advanced CRC [127]. Retinoic acid induced 2 (*RAI2*) methylation serves as an independent poor prognostic marker in CRC by inhibiting the protein kinase B (AKT) signaling pathway and suppressing CRC cell growth *in vitro* and *in vivo* [128]. Frequent hypermethylation of *DIRAS1* in human CRC, regulated by promoter region methylation, positions it as a potential marker for poor prognosis [129]. Suppressor of variegation 3-9 homolog 2 (*SUV39H2*), a lysine methyltransferase, predicts CRC prognosis and promotes malignant phenotypes by tri-methylating the slit guidance ligand 1 (*SLIT1*) promoter [130]. *ZNF331*, a frequently methylated transcriptional repressor in CRC, has emerged as a potential marker for CRC detection with high specificity (98%) and sensitivity (71%) [98]. Vedeld et al. [131] further confirmed ZNF331 methylation in CRC, indicating a poor prognosis.

## Relationship between DNA methylation and metabolism in CRC

The past few years have witnessed a burgeoning interest in the metabolic reprogramming of tumors during their malignant transformation. Notably, cancer-related metabolic changes are not merely responses to signals for cell proliferation or the survival of damaged mitochondria; rather, they result from oncogene-directed metabolic reprogramming [132, 133]. Numerous studies have established a close relationship between metabolic alterations and epigenetic changes, some confirming the influence of metabolic reprogramming on DNA methylation in CRC [132–135].

The tricarboxylic acid (TCA) cycle, central to cell metabolism, plays a pivotal role in both catabolic and anabolic processes [134]. Metabolites from the TCA cycle, including α-ketoglutarate, succinic acid, and fumaric acid, may impact carcinogenic signaling, and TCA cycle disruption can directly influence the epigenome [135]. In CRC, mutations in fumarate hydrase or succinate dehydrogenase lead to fumarate and succinate enrichment, influencing DNA demethylase through competitive α-ketoglutarate inhibition. For instance, in CRC cells expressing activated KRAS, glutaminase, and SLC25A22 promote succinate accumulation, resulting in increased DNA methylation, WNT/β-catenin signaling activation, upregulated leucine rich repeat containing G protein-coupled receptor 5 (*LGR5*) expression, enhanced proliferation, acquisition of stem cell features, and resistance to 5-fluorouracil [96].

Furthermore, studies have indicated that epigenetic processes during aging are disrupted in the normal colon of individuals at high CRC risk, aligning with the age-related incidence of CRC. Recent findings, however, revealed an increase in CRC cases among the young, with obesity emerging as a key factor [136]. The highly responsive epigenetic mechanism to metabolic cues, dependent on intermediate metabolites, is closely linked to obesity [137]. Metabolic disturbances associated with obesity in colonic cells result in widespread DNA methylation alterations, particularly in regulatory regions. Metabolic abnormalities triggered by obesity induce DNA methylation changes promoting CRC onset and progression, with adipose tissue playing a significant role. For instance, low 25-hydroxyvitamin D (25(OH)D) levels and reduced expression of the vitamin D receptor (VDR) in CRC may modify DNA methylation in adipose tissue, subsequently promoting inflammation. Obesity-induced DNA methylation alterations affect long-chain fatty acid oxidation-related metabolism in young mice, potentially promoting genetic changes that increase susceptibility to tumor development with age [136]. An expanding body of research underscores the critical role of metabolic reprogramming in methylation processes in CRC.

Methylation changes can be induced by aging, dietary, life habit, and environmental factors, and some of these factors are strongly associated with metabolism. Nutritional factors of the diet can influence epigenetic mechanisms [138]. DNA metabolism and synthesis of methyl donors require B vitamins, methionine, and S-adenosylmethionine for maintenance of DNA methylation [139, 140]. Research has shown that vitamin B12 is closely linked to methionine metabolism, a crucial component of DNA methylation reactions. Additionally, there is evidence suggesting an association between vitamin B12 and DNA methylation and insulin metabolism [141]. Consequently, serum vitamin B12 may serve as an important biomarker for CRC treatment through its influence on DNA methylation. A study examined the interaction of lifestyle factors with age-dependent increases in methylation through log-linear multivariate regression and linked it to the role of hypermethylation as a modifier in CRC. The result shows that lifestyle modulates age-associated DNA methylation change in the colonic epithelium and thereby impacts the evolution of cancer methylomes [142].

Smoking and alcohol consumption increase the risk of developing CRC, which poses a significant global health concern. A particular correlation that was observed was between cigarette smoking and the risk of colon cancer, but only in a subset of tumors that were *CIMP* high and *BRAF* wild type, or *CIMP* high and *BRAF* mutation, along with KRAS wild type. Quitting smoking can offer protection against oncogenic pathways associated with DNA methylation that lead to *CIMP*-high CRC. Zhou et al. [143] evaluated the relationship between alcohol consumption and CRC risk, suggesting that alcohol’s pathogenic effect on CRC may be partially attributed to DNA methylation regulation of CRC associated 1 (*COLCA1*)/*COLCA2* gene expression. However, the relationship between changes in DNA methylation induced by environmental factors and CRC has been under-researched. Although several studies have established a link between behavioral issues such as posttraumatic stress disorder, depression, post-traumatic growth, resilience, and DNA methylation [144, 145].

## Conclusions

CRC is characterized by distinct genetic and epigenetic alterations. The intricate interplay of genetic, epigenetic, and epitranscriptomic changes contribute to cancer occurrence and progression. In CRC, epigenetic modifications often precede genetic alterations in driving malignant transformation. Aberrant epigenetic changes are early events in carcinogenesis, evolving throughout tumor development and metastasis, suggesting specific epigenetic modifications as promising diagnostic and prognostic biomarkers.

While colonoscopy remains the gold standard for CRC detection, its invasive nature and associated risks have prompted the popularity of alternative screening tests. However, technical challenges, inconsistent methodologies across studies, and the relatively low yield of epigenetic material in samples.

To facilitate clinical use, standardization of biomarkers and assays according to tumor type is essential. Patient selection based on tumor stage and biomarkers can improve response rates. Optimization of combination strategies with cytotoxic drugs, immunotherapy, or radiotherapy based on molecular tumor subtype, pharmacodynamics, and expected adverse effects can enhance efficacy and avoid toxicity. With the emergence of personalized therapies, further studies are needed to elucidate the relationship between individual genetic and epigenetic alterations, providing a pathway-driven basis for selecting optimal therapeutic strategies.

Importantly, the discussed epigenetic modifications do not act in isolation; they finely regulate complex regulatory networks and foster crucial interactions. The strong correlation between DNA methylation and histone methylation and ongoing research revealing intricate molecular interactions between these epigenetic marks suggest their dysregulation plays a prominent role in various human cancers, including CRC. Though detailed mechanisms are under investigation, current evidence suggests close crosstalk between DNA and histone methylation contributing to cancer initiation and progression promising deeper insights into CRC research and disease understanding.

The field of metabolic reprogramming of cancer cells during malignant transformation has garnered much attention. Numerous studies have shown that abnormal methylation modifications of specific genes, repetitive sequences, and CpG islands are all closely linked to tumorigenesis. A deeper comprehension of genomic methylation has emerged, as well as a realization of the close correlation between metabolic reprogramming and epigenetic modifications, and how their abnormal crosstalk impacts tumors. Therefore, further clarification of their connections is imperative and crucial for more effective cancer therapy.

## Abbreviations

APC: adenomatosis polyposis coli
AUC: area under the curve
BCAT1: branched chain amino acid transaminase 1
BMP3: bone morphogenetic protein 3
CEA: carcinoembryonic antigen
cfDNA: cell-free DNA
CIMP: CpG island methylator phenotype
CRC: colorectal cancer
ctDNA: circulating tumor DNA
CXCR4: C-X-C motif chemokine receptor 4
DNMTs: DNA methyltransferases
FBN1: fibrillin 1
HDAC: histone deacetylase domain protein
HPP1: hyperpigmentation progressive 1
IKZF1: IKAROS family zinc finger 1
ITGA4: integrin subunit alpha 4
LINE-1: long interspersed element-1
MGMT: O^6^-methylguanine DNA methyltransferase
MLH1: MutL homolog 1
MSI: microsatellite instable
MSS: microsatellite stable
NDRG4: NDRG family member 4
PCDHB3: protocadherin beta 3
PRIMA1: proline rich membrane anchor 1
RASSF2A: ras association domain family member 2a
SDC2: syndecan 2
SEPT9: septin 9
SFRP1: secreted frizzled related protein-1
SLC25A22: solute carrier family 25 member 22
SNCA: synuclein, alpha
SPG20: spastic paraplegia 20
TCA: tricarboxylic acid
TFPI2: tissue factor pathway inhibitor-2
TSG: tumor suppressor gene
UHRF1: ubiquitin like with PHD and ring finger domains 1
VIM: vimentin
WIF1: Wnt inhibitory factor-1
ZNF304: zinc finger protein 304

## Declarations

### Copyright

© The Author(s) 2024.
